# Results of streamlining TAVR procedure towards a minimalist approach: a single center experience in Taiwan

**DOI:** 10.1038/s41598-023-46475-4

**Published:** 2023-11-06

**Authors:** Tsung-Yu Ko, Hsien-Li Kao, Yi-Chang Chen, Chih-Fan Yeh, Ching-Chang Huang, Ying-Hsien Chen, Chih-Yang Chan, Lung-Chun Lin, Ming-Jiuh Wang, Yih-Sharng Chen, Mao-Shin Lin

**Affiliations:** 1 Division of Cardiology, Department of Internal Medicine and Cardiovascular Center, National Taiwan University Hospital, Taipei, Taiwan; 2 Graduate Institute of Clinical Medicine, National Taiwan University College of Medicine, Taipei, Taiwan; 3 Department of Medical Imaging, National Taiwan University Hospital, Taipei, Taiwan; 4 Department of Surgery, National Taiwan University Hospital, Taipei, Taiwan; 5 Department of Anesthesiology, National Taiwan University Hospital, Taipei, Taiwan

**Keywords:** Cardiology, Medical research

## Abstract

Trans-femoral transcatheter aortic valve replacement (TF-TAVR) performed under conscious sedation (LACS) is not yet become routine practice in Taiwan. We aimed to compared the results between patients received general anesthesia (GA) versus LACS. Our cohort was divided into 3 groups: initial 48 patients received TF-TAVR under routine GA (GA group), subsequent 50 patients under routine LACS (LACS group 1), and recent 125 patients under LACS (LACS group 2). The baseline, procedural characteristics and all outcomes were prospectively collected and retrospectively compared. From Sep 2010 to July 2019, a total of 223 patients were included. The procedure time (157.6 ± 39.4 min vs 131.6 ± 30.3 vs 95.2 ± 40.0, < 0.0001), contrast medium consumption (245.6 ± 92.6 ml vs 207.8 ± 77.9 vs 175.1 ± 64.6, < 0.0001), length of intensive care unit (2 [1–5] days vs 2 [1–3] vs 1 [1–1], P = 0.0001) and hospital stay (9 [7–13] days vs 8 [6–11] vs 6 [5–9], P = 0.0001) decreased significantly with LACS, combined with a trend of less hospital acquired pneumonia (12.5% vs 6.0% vs 5.6%, P = 0.427). 1-year survival rate were also different among 3 groups (83.3% vs 90.0% vs 93.6%, P = 0.053). In our single center experience, a “minimalist” approach of TF-TAVR procedure resulted in less medical resources usage, along with more favorable clinical outcomes.

## Introduction

Since the introduction of transcatheter aortic valve replacement (TAVR), the procedures have been performed under general anesthesia (GA). With increasing operator experience, advancement of transcatheter valve systems, and economic considerations, streamlining transfemoral TAVR (TF-TAVR) procedure from GA to local anesthesia or conscious sedation (LACS) has been considered and advocated. The issues of GA included: potential discomfort and complications of tracheal intubation and mechanical ventilation, hemodynamic compromise induced by anesthetic agents and subsequent need for catecholamine use. These may be avoided by LACS, but aspiration may occur because of a non-protected airway. The absence of intra-procedural trans-esophageal echocardiography (TEE) under LACS may also lead to higher incidence of paravalvular leakage (PVL)^1^. Previous registries reported the benefits of LACS with shorter procedure times, intensive care unit (ICU) and hospital stays, and lower 30-days mortality^2–6^. However, experiential heterogeneity would unavoidably confound the results because most centers started TAVR procedure with GA, and then switched to LACS with increasing experience. Otherwise, differences in clinical practice and patient characteristics may result in different results, and the benefit of LACS were not found in a recent randomized trial^7^. The economic effects of “minimalist” approach of TF-TAVR procedure were also unknown, and may be dependent on the local reimbursement system. Herein, we report our single center experience in Taiwan in streamlining TF-TAVR procedure from GA to LACS. We also divided the LACS population into 2 groups by time sequence to clarify the effect of experience and background heterogeneity on TAVR outcome.

## Materials and methods

### Patients population and data collection

**Figure 1:**
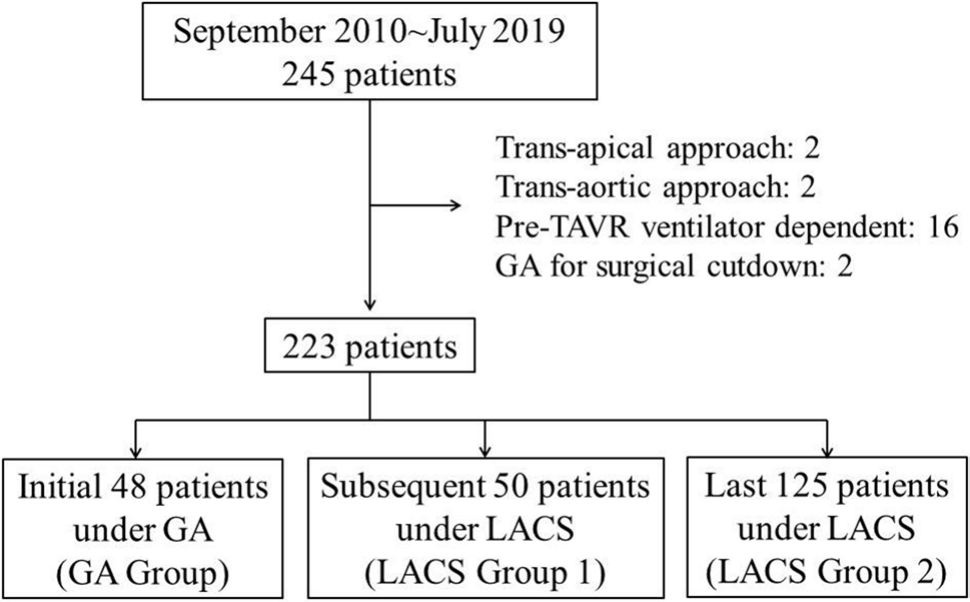
Study flow-chart outlining recruitment and grouping for patients having either general anesthesia (GA) or local anesthesia/conscious sedation (LACS) for a transcatheter aortic valve replacement (TAVR) procedure.

245 consecutive patients with severe symptomatic aortic stenosis underwent TF-TAVR in National Taiwan University Hospital from September 2010 to June 2019. 223 patients were included in the final analysis, according to the flowchart depicted in Fig. 1. TF-TAVR was performed under GA routinely from September 2010 to July 2014. After July 2014, we switched to LACS as routine practice. Therefore, among the 223 patients analyzed, 48 patients received TF-TAVR procedure under GA (as GA group). To minimize the impact of TF-TAVR experience in procedural parameters and outcomes, we then further divided the 175 LACS patients into 2 groups: initial 50 LACS patients LACS group 1 (from September 2014 to March 2016), and the subsequent 125 patients as LACS group 2 (from April 2016 to July 2019). Baseline patient characteristic, procedural variables, and outcomes were prospectively collected. The medical fees of the index hospitalization (including medical fee of TAVR procedure, medication, hospitalization, and devices other than transcatheter heart valve) were also collected.

### Ethical approval statement

All patients had signed informed consent, and the study was approved by the institutional review board of National Taiwan University Hospital. All methods were carried out in accordance with relevant guidelines and regulations.

### Treatment and follow-up

GA was performed with endotracheal intubation and mechanical ventilator support. Variable combination of intravenous anesthetic agents (sevofluorane or propofol), muscle relaxants (rocuronium or cisatracurium), and ultra-short-acting opioid (remifentanil). Proper anesthesia level was maintained to allow immediate extubation at the end of TF-TAVR procedure in the operating room. Assessment of peri-procedural TAVR results by TEE was performed in GA group in addition to the angiographic and hemodynamic evaluation.

In LACS patients, local analgesics were applied by the operators. Conscious sedation was performed by an anesthesiologist with a continuous infusion of either midazolam, dexmedetomidine, or remifentanil. The medication was titrated to induce moderate sedation, during which patients respond purposefully to verbal commands, either alone or accompanied by light tactile stimulation. Post-TAVR assessment was based on angiographic and hemodynamic, and in some cases, transthoracic echocardiographic evaluation in LACS patients.

Clinical follow-up was performed at discharge, 30 days, 6 and 12 months post TAVR. Definitions of procedure results were in accordance with the Valve Academic Research Consortium (VARC) consensus^8–10^.

### Statistical analysis

Continuous variables were presented as mean ± standard deviation (SD) or mean with interquartile range (IQR). ANOVA were used to compared 3 groups, and post hoc-analysis were performed between each 2 groups if ANOVA showed difference with an adjusted p value according to Bonferroni method. Categorical variables were presented as percentage and compared with either chi-square test or Fisher exact test. Cumulative rate of death was presented with Kaplan Meier method and assessed with log-rank test. Cox regression analysis were used to compared the 1-years survival of LACS1 vs. GA, and LACS2 vs. GA respectively, potential confounders (P value < 0.1 in Table 1) were adjusted. All tests were 2-sided, and P value < 0.05 was considered as statistically significant. The statistical analyses were performed using STATA version 14.2 (STATA Corp, Texas, USA).

**Table 1:** Baseline characteristics.

	GA group (N = 48)	LACS group 1 (N = 50)	LACS group 2 (N = 125)	P value
Age (years)	83.1 ± 4.4	80.7 ± 8.8	81.0 ± 7.2	0.171
Male sex	24 (50%)	28 (56%)	52 (41.6%)	0.211
BMI (kg/m^2^)	23.1 ± 3.7	23.0 ± 3.5	24.2 ± 5.1	0.198
Bicuspid	1 (2.1%)	9 (18%)*	13 (10.6%)	0.111
STS mortality score	5.9 (4.1–8.7)	5.9 (3.7–8.8)	3.7 (2.1–6.4)	< 0.001
NYHA Fc III and IV	39 (81.2%)	45 (90.0%)	98 (78.4%)	0.462
Diabetes mellitus	16 (33.3%)	14 (28.0%)	46 (36.8%)	0.462
Hypertension	38 (79.2%)	24 (48.0%)*	80 (64.0%)	0.031
Atrial fibrillation	12 (25.0%)	8 (16.0%)	22 (17.6%)	0.454
COPD	7 (14.6%)	8 (16.0%)	6 (4.8%)*†	0.028
CKD	16 (33.3%)	18 (36.0%)	43 (34.4%)	0.961
CAD	23 (47.9%)	21 (42.0%)	54 (43.2%)	0.814
Previous MI	8 (16.7%)	1 (2.0%)*	6 (4.8%)*	0.011
Previous CABG	2 (4.2%)	4 (8.0%)	4 (3.3%)	0.392
PPM	6 (12.5%)	1 (2.0%)	6 (4.9%)	0.100
PAOD	7 (14.6%)	6 (12.0%)	19 (15.5%)	0.843
LVEF (%)	64.2 ± 13.3	67.8 ± 12.2	65.3 ± 12.4	0.315
AV max PG (mmHg)	84.3 ± 28.9	77.8 ± 27.5	71.6 ± 27.5*	0.027
AV area (cm^2^)	0.65 ± 0.19	0.69 ± 0.17	0.78 ± 0.18*^†^	< 0.0001

For continuous variables, values were mean ± SD.

*GA* general anesthesia, *LACS* local anesthesia or consciousness sedation, *AV* aortic valve, *BMI* body mass index, *CABG* coronary artery bypass graft, *CAD* coronary artery disease, *CKD* chronic kidney disease, *COPD* chronic obstructive lung disease, *GA* general anesthesia, *LACS* local anesthesia or conscious sedation, *LVEF* left ventricular ejection fraction, *MI* myocardial infarction, *NYHA Fc* New-York heart association functional class, *PG* pressure gradient, *PPM* permanent pacemaker, *STS* society of thoracic surgeon.

*Adjusted P < 0.05 as compared to GA group.

^†^Adjusted P < 0.05 as compared to LACS group 1.

## Results

### Study population

The baseline characteristics of 223 patients were summarized in Table 1. Society of Thoracic Surgeons (STS) mortality score was lower in LACS group 2 (GA group vs LACS group 1 vs LACS group 2: 5.9 (4.1–8.7) vs 5.9 (3.7–8.8) vs 3.7 (2.1–6.4), P < 0.001). Lower incidence of chronic obstructive pulmonary disease (14.6% vs 16.0% vs 4.8%, P = 0.028) and larger pre-TAVR aortic valve area (0.65 ± 0.19 cm^2^ vs 0.69 ± 0.17 vs 0.78 ± 0.18, P < 0.0001) was also observed in LACS group 2. Higher incidence of hypertension (79.2% vs 48.0% vs 64.0%, P = 0.031), previous myocardial infarction (16.7% vs 2.0% vs 4.8%, P = 0.011) were seen in GA group. There was no difference in age and left ventricular systolic function among 3 groups.

### Procedural variables

The procedural variables and results were presented in Table 2. TF-TAVR was performed via groin surgical cutdown in the first 30 patients (all in GA group), and then percutaneously with access pre-closure devices, either by 2 ProGlide (Abbott Vascular Inc., Santa Clara, Ca, USA) or combination of 1 ProGlide and 1 Angio-Seal (St. Jude Medical, St. Paul, MN, USA)^9^. Procedure time (GA group vs LACS group 1 vs group 2: 157.6 ± 39.4 min vs 131.6 ± 30.3 vs 95.2 ± 40.0, < 0.0001), contrast medium consumption (245.6 ± 92.6 ml vs 207.8 ± 77.9 vs 175.1 ± 64.6, < 0.0001) were significantly reduced in LACS group 1 and 2. The percentage of more than moderate PVL post-TAVR was similar in GA, LACS group 1 and 2. The device success rate was higher in LACS group 2, as compared to that in GA group. (92.8% vs 83.3%, P = 0.003).

**Table 2:** Procedural variables and results.

	GA group (N = 48)	LACS group 1 (N = 50)	LACS group 2 (N = 125)	P value
Surgical cutdown	30 (62.5%)	0 (0%)	0 (0%)	< 0.0001
Valve type				< 0.0001
CoreValve or EvolutR	48 (100.0%)	41 (82.0%)	53 (42.4%)	
Lotus	0	7 (14.0%)	3 (2.4%)	
Sapien XT or Sapien 3	0	2 (4.0%)	64 (51.2%)	
Portico	0	0	5 (4.0%)	
Contrast volume (ml)	245.6 ± 92.6	207.8 ± 77.9	175.1 ± 64.6*^†^	< 0.0001
Procedure time (min)	157.6 ± 39.4	131.6 ± 30.3*	95.2 ± 40.0*^†^	< 0.0001
Fluoroscopy time (min)	22.0 (15.4–24.1)	24.8 (19.1–37.1)	21.3 (16.7–25.8)	0.069
Major vascular complication	1 (2.1%)	0 (0.0%)	4 (3.2%)	0.427
More than moderate PVL	4 (8.3%)	5 (10.0%)	6 (4.8%)	0.402
Device success	40 (83.3%)	45 (90.0%)	116 (92.8%)*	0.174

Continuous variables were mean ± SD.

*GA* general anesthesia, *LACS* local anesthesia or conscious sedation, *PVL* paravalvular leakage.

*Adjusted P < 0.05 as compared to GA group.

^†^Adjusted P < 0.05 as compared to LACS group 1.

### In-hospital results and clinical outcomes

**Figure 2:**
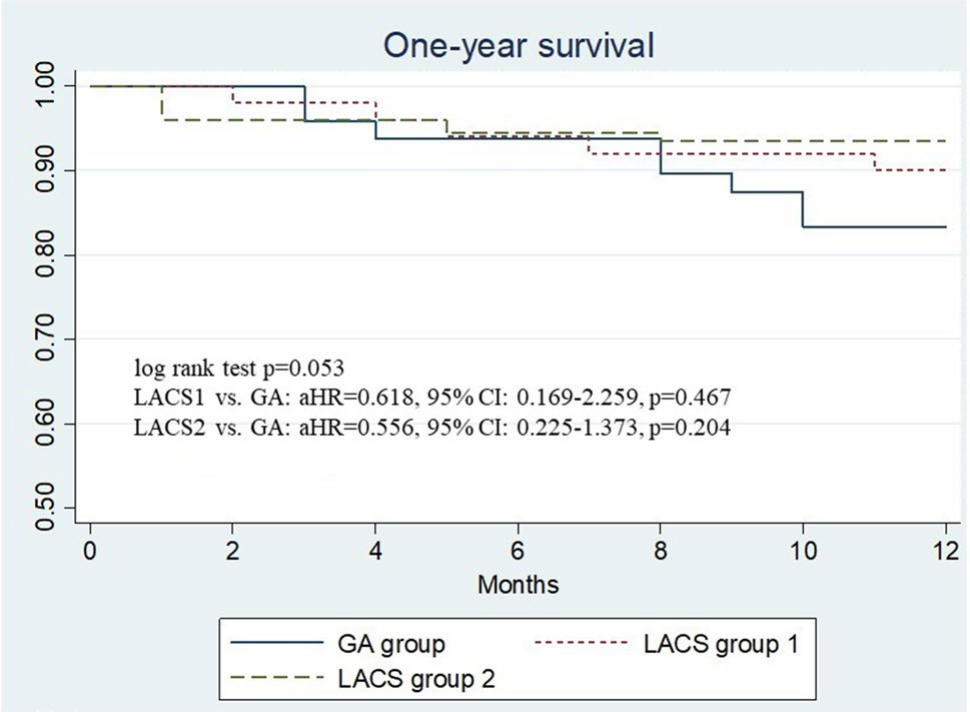
Kaplan-Meier curve of 1-year survival between groups. *GA* general anesthesia, *LACS* local anesthesia or conscious sedation, *aHR* adjusted hazard ratio, *CI* confidence interval

The in-hospital results and clinical outcomes were presented in Table 3. The length of ICU stay were different either among groups (GA group vs LACS group 1 vs group 2: 2 [1–5] days vs. 2 [1–3] vs. 1 [1], P = 0.0001) or comparing each of the 2 groups (GA group vs LACS group 1, P = 0.0009; GA group vs LACS group 2, P < 0.0001. LACS group 1 vs group 2, P = 0.0001). The hospital stay (9 [7–13] days vs. 8 [6–11] vs. 6 [5–9], P = 0.0001) were shorter in LACS group 1 and 2. There was a trend of less in-hospital complications including hospital acquired pneumonia (HAP) and gastro-intestinal (GI) bleeding in LACS group 1 and 2. Medical fees during hospitalization (267,906 [193,116–380,331] NT$ vs 209,398 [151,889–314,497] vs 190,945 [111,713–296,163], P = 0.0079) were significantly lower in LACS group 1 and 2. There was no statistical difference in the 30-days mortality observed among groups (0% vs. 0% vs. 3.2%, P = 0.42). Figure 2 is the Kaplan–Meier survival curve, showing borderline statistical difference in the 1-year survival among groups (83.3% vs. 90.0% vs. 93.6%, log-rank test P = 0.053). The adjusted hazard ratio (aHR) of LACS1 vs. GA was 0.618 (95% confidence interval (CI) 0.169–2.259, P = 0.467); aHR of LCAS2 vs. GA was 0.556 (95% CI 0.225–1.373, P = 0.204).

**Table 3:** In-hospital results and clinical outcomes.

	GA group (N = 48)	LACS group 1 (N = 50)	LACS group 2 (N = 125)	P value
ICU stay (days)	2 (1–5)	2 (1–3)*	1 (1–1)*^†^	0.0001
Hospital stay (days)	9 (7–13)	8 (6–11)	6 (5–9)*^†^	0.0001
Hospital acquired pneumonia	6 (12.5%)	3 (6.0%)	7 (5.6%)	0.427
In-hospital GI bleeding	5 (10.4%)	2 (4.0%)	4 (3.2%)	0.235
In-hospital AKI	4 (8.3%)	2 (4.0%)	13 (10.4%)	0.66
In-hospital stroke	1 (2.1%)	1 (2.0%)	2 (1.6%)	0.829
Post-TAVR PPM	10 (20.8%)	11 (22.0%)	17 (13.6%)	0.147
Medical fee reimbursed during hospitalization (NT dollars)^a^	267,906 (193,116–380,331)	209,398 (151,889–314,497)*	190,945 (111,713–296,163)*	0.0079
30-day mortality	0 (0.0%)	0 (0.0%)	4 (3.2%)	0.42

ICU and Hospital stay, medical fee reimbursed during hospitalization were presented with median and interquartile range.

*GA* general anesthesia, *LACS* local anesthesia or consciousness sedation, *ICU* intensive-care unit, *GI bleeding* gastro-intestinal bleeding, *AKI* acute kidney injury, *TAVR* transcatheter aortic valve replacement, *PPM* permanent pacemaker, *NT dollars* New-Taiwan dollars.

*Adjusted P < 0.05 as compared to GA group.

^†^Adjusted P < 0.05 as compared to LACS group 1.

^a^Including medical fee of TAVR procedure, medication, hospitalization, and devices other than transcatheter heart valve.

## Discussion

**Figure 3:**
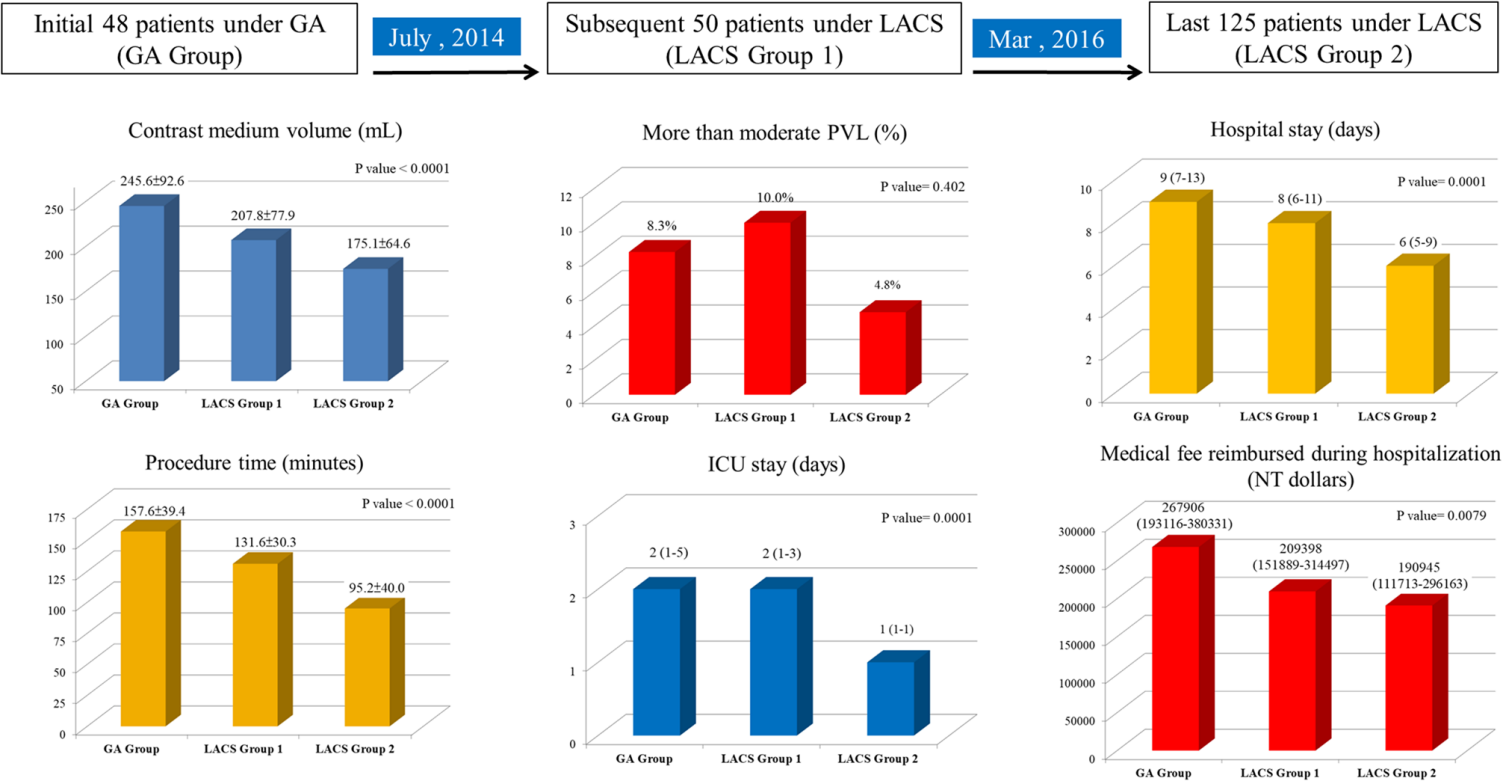
Central figure summarizing the main results. *GA* general anesthesia, *ICU* intensive care unit, *LACS* local anesthesia or conscious sedation, *NT dollars* new Taiwan dollars, *PVL* paravalvular leakage.

The number of TAVR procedures performed under local anesthesia with or without conscious sedation is rising^3,11^. According to Transcatheter Valve Therapy Registry analyzing 120,080 TF-TAVR patients between January 2016 and March 2019 in the U.S., the use of LACS increased substantially from 33 to 64%, but 17% of the U.S. centers continued to use GA exclusively^11^. In Taiwan, TF-TAVR procedures performed under LACS were still less than 50%. Which anesthetic management is more favorable for TF-TAVR is now a subject of intense debate, leading to a considerable variation in clinical practice. Based on the results of this single center study, LACS is not only a feasible alternative to conventional GA for TF-TAVR, but also results in less resource usage, comparable with the observations in previous registries^4–6,11^ (Fig. 3). Practicing this “minimalist” approach, improved clinical outcomes with very limited 30-days (4/175, 2.2%) and 1-year mortality (13/175, 7.4%) could be achieved in a center with an annual TAVR case number less than 100, we think the benefit of LACS may be easier to achieve and more cost-effective in centers with larger amount of TF-TAVR case.

Most centers started TAVR procedure with GA. But with increasing experience and familiarity with devices, operators tend to switch from GA to LACS^12^. The results of previous registries^2–6,11–13^ investigating LACS versus GA in TF-TAVR were therefore unavoidably confounded by experiential heterogeneity^2^. Despite statistical maneuvers such as propensity matching, these results may merely reflect differences in patient selection and learning curve over time, instead of the switching from GA to LACS itself. To clarify the effect of TAVR experience and background heterogeneity, we divided 175 LACS TF-TAVR patients in the present analysis into 2 groups. With similar experiential and risk background (STS mortality score: GA group vs LACS group 1 = 7.4 ± 4.6 vs 6.8 ± 4.4, P = 0.548) as compared to GA group, significantly less contrast medium consumption (245.6 ± 92.6 ml vs 207.8 ± 77.9 ml, P = 0.05), shorter procedure time (157.6 ± 39.4 min vs 131.6 ± 30.3 min, P = 0.001), ICU stay (2 [1–5] days vs 2 [1–3] days, P = 0.009) and less in-hospital medical fee (267,906 [193,116–380,331] vs 209,398 [151,889–314,497], P = 0.0297) were still observed in LACS group 1. Although the effect of improvement in technical skills can never be ruled out, the results could still be attributed to the “minimalist” approach: simplification of the anesthetic strategies and access pre-closure. The importance of reducing contrast medium usage is potentially lowering the risk of contrast-induced nephropathy, especially for the elderly with multiple comorbidities such as chronic kidney disease. The faster procedure sometimes represents the less complicated peri-procedural course. It will lead to less complicated post-procedural course, and may result in a significant reduction in health care expenses, which already suggested by a previous study^14^.

Tracheal intubation with mechanical ventilation, indwelling urinary tract catheters, hemodynamic instability, and prolonged hospitalization associated with GA may increase the risk of infection. Interestingly, TAVR peri-procedural infections were never reported in previous single center reports^15,16^, or registry studies^4–6,11,13,17^ comparing of anesthetic managements. Peri-procedural infection is not part of the endpoints in Valve Academic Research Consortium (VARC), except for infective endocarditis after TAVR^8,9^. In the SOLVE-TAVI trial, infections requiring antibiotic treatment occurred in one-fifth of the patients, mainly attributable to pneumonia and urinary tract infection. The incidences of overall infections were reported similar between GA & LACS groups, but the risk of pneumonia was not mentioned specifically^7^. In our cohort, the incidence of hospital acquired pneumonia (HAP) in GA group was 2-times higher than that in LACS group 1 and 2, although not statistically significant due to sample size. Significantly lower HAP rate, however, was seen in LACS group 2 comparing to that in GA group, but this was probably confounded by the different risk profile of the patient populations.

Lower incidence of in-hospital gastro-intestinal (GI) bleeding was also found in the LACS groups in the present study, which was never reported in previous registries or randomized trial comparing GA versus LACS in TAVR. GI bleeding was included in major and minor bleeding complications in VARC definition, and was not counted independently^7,8^. Stanger et al. reported a retrospective single center evaluation of 841 TAVR patients, and overall risk of upper GI bleeding following TAVR was found to be 2.0% (17/841)^18^. Patients on triple antithrombotic therapy are at highest risk for severe upper GI bleeding. Upper endoscopy evaluation or treatment was done in 12 patients, and the most common lesion was a distal esophageal or gastroesophageal junction ulceration with active bleeding. They postulated that the use of intraoperative TEE, which may cause local mechanical and thermal trauma, was the reason for these findings. The mechanism and impact of in-hospital GI bleeding following TAVR should be studied further in the future.

The present study has a number of limitations. Firstly, this is a single center study with a relatively small consecutive cohort. The 2 discussed anesthetic strategies were chosen arbitrarily over time without randomization, and also the baseline characteristics were different between each group which may bias the results. Thus the generalization of our finding was limited. Secondly, because of the chronological nature of the study, the results may be confounded by differences in procedure experience and patient risk profiles, as well as the simultaneous introduction of other technological advances in TAVR, such as newer generation of valve systems. Thirdly, the actual anesthetic agents and dosages applied in LACS or GA were not pre-specified or controlled, and their potential influence on the outcome was difficult to be examined by the present study.

## Conclusion

The present study provided the contemporary results of single center experience in Taiwan in streamlining TAVR procedure towards a “minimalist” approach. It demonstrated the feasibility and safety of TF-TAVR performed using exclusively LACS. The contrast volume, procedure time, ICU and hospital stays were all reduced significantly. There was also a trend of lower incidence of HAP and GI bleeding in patients receiving LACS. All these translated into less in-hospital medical cost without compromised efficacy and a very low 30-day and 1-year mortality. The results demonstrated that LACS is a viable alternative to a traditional GA for TF-TAVR, and should be considered by all institutions performing this procedure in Taiwan.

## Data Availability

The datasets used and/or analysed during the current study available from the corresponding author on reasonable request.
